# Association between serum osmolality and risk of in-hospital mortality in patients with intracerebral hemorrhage

**DOI:** 10.3389/fneur.2024.1410569

**Published:** 2024-08-02

**Authors:** Zhaosuo Hu, Quan Sha

**Affiliations:** ^1^School of Basic Medicine, Anhui Medical University, Hefei, Anhui, China; ^2^Department of Clinical Laboratory, Hefei Hospital Affiliated to Anhui Medical University, Hefei, Anhui, China

**Keywords:** intracerebral hemorrhage, in-hospital mortality, serum osmolality, ICU, blood urea nitrogen to creatinine ratio

## Abstract

**Aim:**

This study aimed to analyze the association between serum osmolality and the risk of in-hospital mortality in intracerebral hemorrhage (ICH) patients.

**Methods:**

In this retrospective cohort study, data of a total of 1,837 ICH patients aged ≥18 years were extracted from the Medical Information Mart for Intensive Care-IV (MIMIC-IV). Serum osmolality and blood urea nitrogen (BUN)-to-creatinine (Cr) ratio (BCR) were used as the main variables to assess their association with the risk of in-hospital mortality in ICH patients after first intensive care unit (ICU) admission using a univariable Cox model. Univariable and multivariable Cox regression analyses were applied to explore the associations between serum osmolality, BCR, and in-hospital mortality of ICH patients. Hazard ratio (HR) and 95% confidence intervals (CIs) were calculated.

**Results:**

The median survival duration of all participants was 8.29 (4.61–15.24) days. Serum osmolality of ≥295 mmol/L was correlated with an increased risk of in-hospital mortality in patients with ICH (HR = 1.43, 95%CI: 1.14–1.78). BCR of >20 was not significantly associated with the risk of in-hospital mortality in ICH patients. A subgroup analysis indicated an increased risk of in-hospital mortality among ICH patients who were women, belonged to white or Black race, or had complications with acute kidney injury (AKI).

**Conclusion:**

High serum osmolality was associated with an increased risk of in-hospital mortality among ICH patients.

## Introduction

Intracerebral hemorrhage (ICH) is a common neurological complication, accounting for approximately 10–20% of all stroke types (1–3). ICH manifests as a rapidly expanding hematoma within the brain tissue, with the potential to extend into the ventricular system and subarachnoid or dural spaces (4). Acute ICH is a medical complication with high mortality, morbidity, and disability rates (5). Evidence from previous studies has revealed that an estimated 10–30 patients per 100,000 persons are affected annually, with 1-year mortality rates as high as 50% in the first 30 days (6). Therefore, accurate prognostic assessment is essential in the clinical management of ICH patients.

Serum osmolality is the serum concentration of ions and particles dissolved in body fluids, reflecting fluid balance and renal function, and is strongly influenced by sodium, potassium, glucose, and urea nitrogen concentrations (7). Dehydration, reflected by high osmolality, is an important risk factor affecting the prognosis of ICU patients (8, 9). The blood urea nitrogen (BUN)-to-creatinine (Cr) ratio (BCR) is clinically considered a simple marker reflecting hydration status (10). However, the BCR is not a specific marker indicating dehydration and may be affected by catabolic status (such as surgery and sepsis) and drug use (11, 12). Direct measurement of plasma osmolality is considered the gold standard to reflect hydration status (13). However, plasma osmolality measurement is not routinely performed, owing to its complexity and high cost. Considering this limitation, serum osmolality calculated by indicators such as sodium, potassium, glucose, and BUN was proposed to replace direct measurement techniques (14). The European Society for Clinical Nutrition and Metabolism (ESPEN) equation has been proven to be highly accurate and has been recommended as a marker for the clinical assessment of dehydration (7, 12). Recent studies have found that serum osmolality calculated by using the ESPEN equation is significantly related to the short-term mortality risk of acute ischemic stroke (15). Moreover, the calculated serum osmolality has a better predictive value for neurological deterioration than BCR for patients hospitalized due to acute ischemic stroke (16). However, BCR also demonstrates outstanding performance in predicting the risk of death in ICH patients (17, 18). Thus, the prognostic value of serum osmolality for ICH still remains unclear.

This study aimed to analyze the association between serum osmolality and the risk of in-hospital mortality in ICH patients based on the Medical Information Mart for Intensive Care-IV (MIMIC-IV) database. The results were compared with those obtained by the BCR. A subgroup analysis was performed in terms of age, gender, and Glasgow Coma Scale (GCS) score.

## Methods

### Study design and population

In this cohort study, data of a total of 2,312 ICH patients aged ≥18 years were identified from MIMIC-IV and extracted. The Medical Information Mart for Intensive Care (MIMIC) database was established in 2003 through funding from Beth Israel Deaconess Medical Center (BIDMC), Massachusetts Institute of Health, the National Institutes of Health Technology, Massachusetts General Hospital, emergency room physicians, critical care physicians, computer science experts, and other professionals in the field of critical care medicine. The MIMIC is recognized as the largest open-source and freely accessible clinical database specifically designed for research on critical care and emergency departments. MIMIC-IV encompassed data collected between 2008 and 2019 (19). Patient records were extracted from the MIMIC-IV database for this study if they met the following criteria: aged ≥18 years old, diagnosed with ICH at ICU admission, and hospitalized in the ICU for at least 24 h. Patients without data on serum osmolality or BCR calculations, those without survival information, or those without data on GCS score were excluded. The requirement of ethical approval for this study was waived by the Institutional Review Board of School of Basic Medicine, Anhui Medical University, because the data were accessed from MIMIC-IV. The need for written informed consent was waived by the Institutional Review Board of School of Basic Medicine, Anhui Medical University, due to the retrospective nature of the study.

### Potential covariates and definitions

Demographic characteristics including age (<65 year or ≥ 65 years); gender (women or men); race (white, Black, other, or unknown); insurance type (Medicare, Medicaid, or other); marital status (married, single, divorced, widowed, or unknown); complications including hypertension, neurological deterioration, acute kidney injury (AKI), and sepsis; Simplified Acute Physiology Score II (SAPSII); Sequential Organ Failure Assessment (SOFA) score; Glasgow Coma Scale (GCS) score (<13 or ≥ 13); Charlson Comorbidity Index (CCI); ICU type [Neuro ICU, surgical Intensive Care Unit (SICU), or other]; treatments including receiving mechanical ventilation, vasopressor, diuretics, mannitol, brain surgery, or cerebral drainage or not; and laboratory data including WBC (K/uL), platelet (K/uL), red cell distribution width (RDW) (%), hemoglobin (g/dL), hematocrit (%), bicarbonate (mEq/L), estimated glomerular filtration rate (eGFR) (mL/min/1.73m^2^); International Normalized Ratio (INR), calcium (mg/dL), chloride (mEq/L), mean arterial pressure (MAP) (mmHg), heart rate (bpm), respiratory rate (insp/min), temperature (°C), oxyhemoglobin saturation (SpO_2_) (%), prothrombin time (PT) (sec), and urine output-24 h (mL) were analyzed as potential covariates.

Acute kidney injury (AKI) was determined based on the Kidney Disease: Improving Global Outcomes (KDIGO) definition. According to this definition, AKI was diagnosed as an increase of 0.3 mg/dL in the serum creatinine (SCr) level within 48 h or an increase in SCr level to 1.5 times of the baseline value, which is known or presumed to have occurred within the previous 7 days, or a urine volume of <0.5 mL/kg/h for 6 h. Sepsis was diagnosed according to the Sepsis-3 criteria (20). Patients with documented or suspected infection and an acute change in total SOFA score of ≥ 2 points were reported to have sepsis. Brain surgery included craniotomy (ICD-9 codes: 0120–0129) or minimally invasive surgery (ICD-9 codes: 0221, 0222, 0139, 0101, 0102, and 0109). Neurological deterioration was defined as a decline in the GCS score by ≥2 points during the ICU stay (21). eGFR _CKD-EPI_ (mL/min/1.73m^2^) = 141 × min (Scr/κ, 1) α × max (Scr/κ, 1) -1.029 × 0.993 age × 1.108 (if woman) × 1.159 (if Black), where κ is 0.7 for women and 0.9 for men, α is −0.329 for women and − 0.411 for men, min indicates the minimum of Scr/κ or 1, and max indicates the maximum of Scr/κ. A GCS score of <13 indicated moderate or severe damage, while a GCS score of ≥13 indicated mild damage (22).

### Main and outcome variables

Serum osmolality and BCR were the main variables. According to the ESPEN-recommended equation, serum osmolality is calculated using the following formula:

Serum osmolality = 1.86× (sodium + potassium) + 1.15 × glucose + BUN+14 (all in mmol/L).

A serum osmolality of ≥295 mmol/L indicated dehydration (12, 23, 24). BCR was calculated as the BUN-to-Cr ratio, and a BCR of >20 indicated dehydration (12, 25).

The in-hospital mortality was the outcome. For participants who survived, the follow-up ended when they were discharged from the hospital. For participants who died during the hospital stay, the follow-up ended at the time of death. This information was recorded by the hospital departments or the Social Security Bureau.

### Statistical analysis

The normality of quantitative data was tested by skewness and kurtosis methods, and the homogeneity of variance was tested by Levene’s test. Normal distribution measurement data were described as mean and standard deviation [mean (±SD)]. Student’s t-test (indicated hereafter as t-test) was used for comparison between groups with the assumption of homogeneity of variance, and Satterthwaite’s t-test (indicated hereafter as t’-test) was used for heterogeneity of variance. Non-normally distributed quantitative data were presented as median and quartiles [M (Q₁, Q₃)], and the Wilcoxon rank-sum test was used for between-group comparison. Categorical data were presented by the number and percentage of cases *n* (%), and the chi-square test or Fisher’s exact test was used for within-group comparison. Missing values were dealt with multiple imputation (Supplementary Table 1). Sensitivity analysis was performed to compare the data before and after imputation, and no significant difference was found (Supplementary Table 2). Serum osmolality and BCR were the main variables used to analyze their association with the risk of in-hospital mortality in ICH patients after first ICU admission using the univariable Cox regression model. Potential covariables with a *p-*value of <0.05 in the univariable Cox regression analysis were considered as confounders to be adjusted. Univariable and multivariable Cox regression analyses were applied to examine the association of serum osmolality and BCR with the in-hospital mortality of ICH patients. Subgroup analyses were performed according to age, sex, ethnicity, GCS score, and AKI. Hazard ratio (HR) and 95% confidence intervals (CIs) were calculated. R (version 4.2.3) was used for missing value interpolation, covariate screening, Cox regression analysis, and subgroup analysis.

## Results

### The baseline characteristics of participants in the survival group or death group

**Figure 1:**
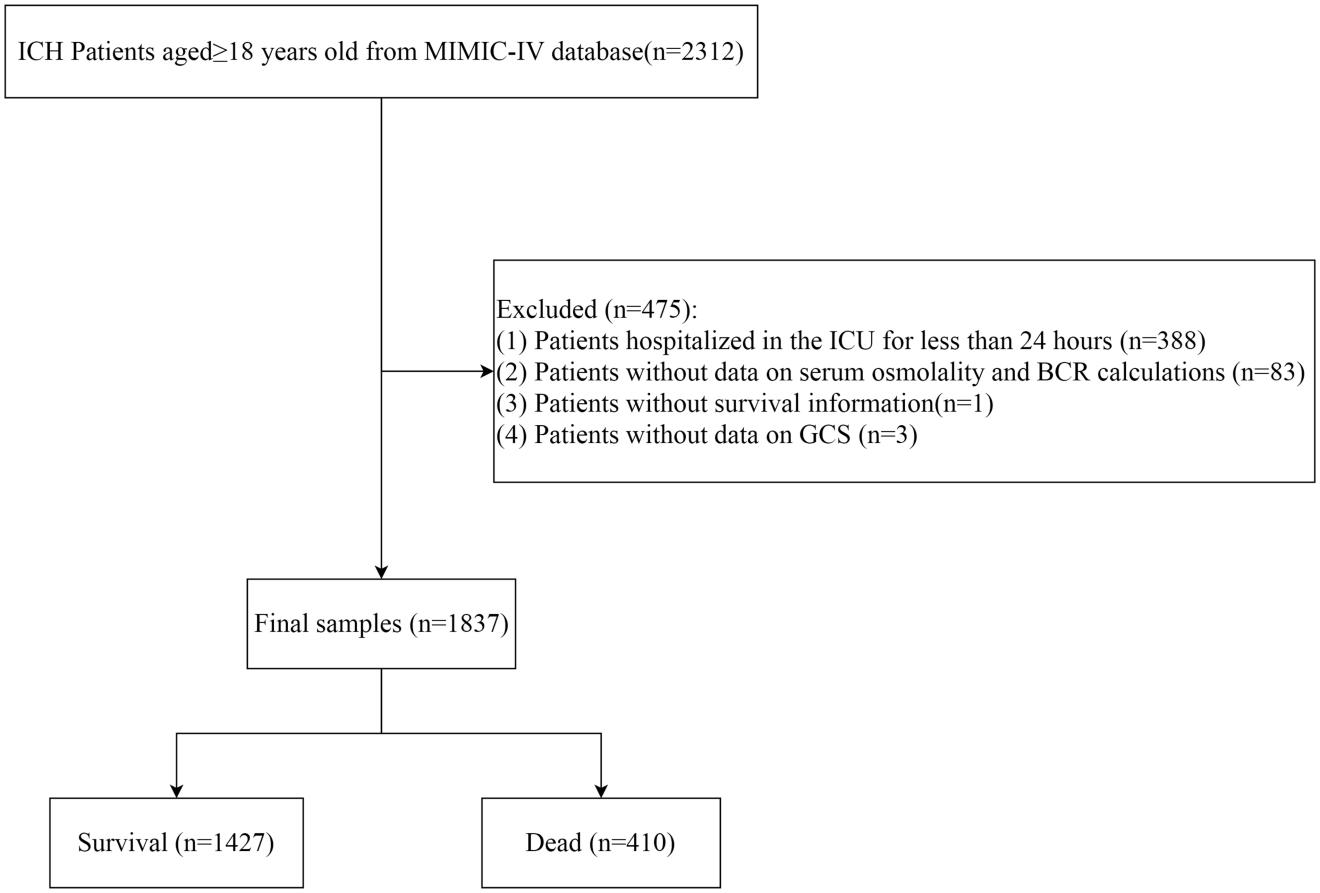
The flowchart of the participants’ selection process.

In total, data of 2,312 ICH patients aged ≥18 years were collected from MIMIC-IV. Among them, patients who were hospitalized in the ICU for less than 24 h were excluded (*n* = 388). Those without data on serum osmolality or BCR calculations (*n* = 83), without survival information (*n* = 1), and subjects without data on GCS (*n* = 3) were excluded. Finally, 1,837 participants were included, consisting of 1,427 survivors and 410 deaths at the end of the follow-up. The screening process is shown in Figure 1.

The median survival time of all participants was 8.29 (4.61–15.24) days. The median serum osmolality in the death group was higher than that in the survival group (298.63 mmol/L vs. 294.85 mmol/L). The percentage of participants with serum osmolality of ≥295 mmol/L in the death group was higher than that in the survival group (64.15% vs. 49.05). The median BCR in the death group was higher than that in the survival group (18.69 vs. 17.50). The percentage of ICH patients with BCR of >20 in the death group was higher than that in the survival group (40.24% vs. 31.39%). More information on the characteristics of survived or dead participants is shown in Table 1.

**Table 1:** The baseline characteristics of participants with ICH.

Variables	Total (*n* = 1,837)	Survival (*n* = 1,427)	Death (*n* = 410)	Statistics	*p*
Survival time, days, M (Q₁, Q₃)	8.29 (4.61–15.24)	9.43 (5.31–16.77)	4.81 (2.22–9.91)	*W* = 408,561	<0.001
Serum osmolality, mmol/L, M (Q₁, Q₃)	295.46 (290.34–301.62)	294.85 (290.22–300.41)	298.63 (291.33–306.16)	*W* = 231890.5	<0.001
Serum osmolality, mmol/L, n (%)				*χ*^2^ = 28.485	<0.001
<295	874 (47.58)	727 (50.95)	147 (35.85)		
≥295	963 (52.42)	700 (49.05)	263 (64.15)		
BCR, M (Q₁, Q₃)	18 (14–22.5)	17.50 (14–22)	18.69 (14.17–23.75)	*W* = 271532.5	0.026
BCR, *n* (%)				*χ*^2^ = 10.823	0.001
≤20	1,224 (66.63)	979 (68.61)	245 (59.76)		
>20	613 (33.37)	448 (31.39)	165 (40.24)		
Sodium, mEq/L, Mean (±SD)	139.08 (±4.77)	139.07 (±4.38)	139.09 (±5.94)	*t*’ = −0.059	0.953
Potassium, mEq/L, M (Q₁, Q₃)	3.9 (3.6–4.3)	3.9 (3.6–4.3)	4 (3.6–4.4)	*W* = 278,188	0.129
Glucose, mg/dL, M (Q₁, Q₃)	129 (107–159)	125 (105–151)	147.5 (121–188)	*W* = 203203.5	<0.001
BUN, mg/dL, M (Q₁, Q₃)	16 (12–22)	16 (12–21)	18 (14–27)	*W* = 224,188	<0.001
Blood creatinine, mg/dL, M (Q₁, Q₃)	0.9 (0.7–1.1)	0.9 (0.7–1.1)	1 (0.7–1.4)	*W* = 238,771	<0.001
Age, years, *n* (%)				*χ*^2^ = 13.683	<0.001
<65	771 (41.97)	632 (44.29)	139 (33.9)		
≥65	1,066 (58.03)	795 (55.71)	271 (66.1)		
Gender, *n* (%)				*χ*^2^ = 0.728	0.393
Woman	833 (45.35)	639 (44.78)	194 (47.32)		
Man	1,004 (54.65)	788 (55.22)	216 (52.68)		
Race, *n* (%)				*χ*^2^ = 35.141	<0.001
White	1,114 (60.64)	900 (63.07)	214 (52.2)		
Black	182 (9.91)	145 (10.16)	37 (9.02)		
Other	218 (11.87)	171 (11.98)	47 (11.46)		
Unknown	323 (17.58)	211 (14.79)	112 (27.32)		
Insurance, *n* (%)				*χ*^2^ = 1.908	0.385
Medicare	820 (44.64)	625 (43.8)	195 (47.56)		
Medicaid	100 (5.44)	80 (5.61)	20 (4.88)		
Other	917 (49.92)	722 (50.6)	195 (47.56)		
Marital Status, *n* (%)				*χ*^2^ = 56.274	<0.001
Married	852 (46.38)	683 (47.86)	169 (41.22)		
Single	381 (20.74)	319 (22.35)	62 (15.12)		
Divorced	109 (5.93)	92 (6.45)	17 (4.15)		
Widowed	228 (12.41)	169 (11.84)	59 (14.39)		
Unknown	267 (14.53)	164 (11.49)	103 (25.12)		
MAP, mmHg, Mean (±SD)	96.44 (±16.99)	96.97 (±16.49)	94.59 (±18.51)	*t*’ = 2.352	0.019
Heart rate, bpm, Mean (±SD)	82.47 (±17.64)	81.85 (±17.04)	84.62 (±19.45)	*t*’ = −2.605	0.009
Respiratory rate, insp/min, Mean (±SD)	18.51 (±5.21)	18.31 (±5.08)	19.19 (±5.56)	*t*’ = −2.870	0.004
Temperature, °C, Mean (±SD)	36.84 (±0.67)	36.85 (±0.58)	36.81 (±0.92)	*t*’ = 0.753	0.452
SpO_2_, %, M (Q₁, Q₃)	98 (96–100)	98 (96–100)	99 (97–100)	*W* = 235,789	<0.001
PT, sec, M (Q₁, Q₃)	12.68 (11.7–14)	12.54 (11.7–13.89)	13.09 (12–14.7)	*W* = 247,156	<0.001
Urineoutput-24 h, mL, M (Q₁, Q₃)	1,650 (1,045–2,370)	1,660 (1,076.5–2,331)	1622.5 (986.25–2586.25)	*W* = 286,764.5	0.542
Cerebrovascular fluid, mL, *n* (%)				*χ*^2^ = 24.282	<0.001
No	1,574 (85.68)	1,254 (87.88)	320 (78.05)		
Yes	263 (14.32)	173 (12.12)	90 (21.95)		
Hypertension, *n* (%)				*χ*^2^ = 0.205	0.651
No	528 (28.74)	406 (28.45)	122 (29.76)		
Yes	1,309 (71.26)	1,021 (71.55)	288 (70.24)		
Neurological deterioration, n (%)				*χ*^2^ = 1.305	0.253
No	1,329 (72.35)	1,042 (73.02)	287 (70)		
Yes	508 (27.65)	385 (26.98)	123 (30)		
AKI, *n* (%)				*χ*^2^ = 33.190	<0.001
No	648 (35.27)	553 (38.75)	95 (23.17)		
Yes	1,189 (64.73)	874 (61.25)	315 (76.83)		
Sepsis, *n* (%)				*χ*^2^ = 36.860	<0.001
No	1,023 (55.69)	849 (59.5)	174 (42.44)		
Yes	814 (44.31)	578 (40.5)	236 (57.56)		
SAPSII, Mean (±SD)	33.43 (±11.73)	31.12 (±10.25)	41.48 (±12.98)	*t*’ = −14.879	<0.001
SOFA, Mean (±SD)	4.35 (±2.97)	3.77 (±2.47)	6.36 (±3.61)	*t*’ = −13.632	<0.001
GCS, *n* (%)				*χ*^2^ = 8.126	0.004
<13	586 (31.9)	431 (30.2)	155 (37.8)		
≥13	1,251 (68.1)	996 (69.8)	255 (62.2)		
CCI, Mean (±SD)	3.53 (±2.28)	3.44 (±2.24)	3.82 (±2.41)	*t* = −3.014	0.003
ICU Type, *n* (%)				*χ*^2^ = 12.157	0.002
Neuro ICU	627 (34.13)	514 (36.02)	113 (27.56)		
SICU	1,009 (54.93)	769 (53.89)	240 (58.54)		
Other	201 (10.94)	144 (10.09)	57 (13.9)		
Mechanical ventilation, *n* (%)				*χ*^2^ = 80.698	<0.001
No	583 (31.74)	528 (37)	55 (13.41)		
Yes	1,254 (68.26)	899 (63)	355 (86.59)		
Vasopressor, *n* (%)				*χ*^2^ = 99.302	<0.001
No	1,597 (86.94)	1,301 (91.17)	296 (72.2)		
Yes	240 (13.06)	126 (8.83)	114 (27.8)		
Diuretic, *n* (%)				*χ*^2^ = 11.096	0.001
No	1,745 (94.99)	1,369 (95.94)	376 (91.71)		
Yes	92 (5.01)	58 (4.06)	34 (8.29)		
Mannitol, *n* (%)				*χ*^2^ = 56.671	<0.001
No	1,724 (93.85)	1,372 (96.15)	352 (85.85)		
Yes	113 (6.15)	55 (3.85)	58 (14.15)		
Brain surgery, *n* (%)				*χ*^2^ = 0.495	0.482
No	1,756 (95.59)	1,361 (95.37)	395 (96.34)		
Yes	81 (4.41)	66 (4.63)	15 (3.66)		
WBC, K/uL, M (Q₁, Q₃)	10.2 (7.9–13.1)	9.8 (7.7–12.7)	11.8 (9.22–14.57)	*W* = 221,869.5	<0.001
Platelet, K/uL, Mean (±SD)	213.13 (±85.12)	217.50 (±83.71)	197.94 (±88.30)	*t* = 4.118	<0.001
RDW, %, Mean (±SD)	14.21 (±1.67)	14.08 (±1.57)	14.64 (±1.91)	*t*’ = −5.446	<0.001
Hemoglobin, g/dL, Mean (±SD)	12.08 (±2.04)	12.19 (±1.97)	11.71 (±2.22)	*t*’ = 3.913	<0.001
Hematocrit, %, Mean (±SD)	36.31 (±5.82)	36.57 (±5.60)	35.38 (±6.43)	*t*’ = 3.417	0.001
Bicarbonate, mEq/L, Mean (±SD)	23.44 (±3.61)	23.62 (±3.40)	22.80 (±4.19)	*t*’ = 3.650	<0.001
eGFR, mL/min/1.73m^2^, Mean (±SD)	81.70 (±27.04)	84.63 (±25.76)	71.50 (±28.90)	*t*’ = 8.304	<0.001
INR, M (Q₁, Q₃)	1.12 (1.1–1.3)	1.1 (1.1–1.26)	1.2 (1.1–1.35)	*W* = 250,128.5	<0.001
Calcium, mg/dL, Mean (±SD)	0.53 (±0.17)	0.53 (±0.16)	0.56 (±0.21)	*t*’ = −2.360	0.019
Chloride, mEq/L, Mean (±SD)	103.47 (±5.32)	103.50 (±4.99)	103.37 (±6.35)	*t*’ = 0.377	0.707

SD, Standard Deviation; M: Median; Q₁, 1st quartile; Q₃, 3rd quartile; t, Student’s t test; t’, Satterthwaite t test; W, Wilcoxon rank-sum test; χ^2^, Chi-square test; −, Fisher’s exact test; ICH, Intracerebral hemorrhage; BCR, Blood uric acid-to-creatinine ratio; BUN, Blood urea nitrogen; MAP, Mean arterial pressure; SpO_2_, Oxyhemoglobin saturation; PT, Prothrombin time; AKI, Acute kidney injury; SAPSII, Simplified Acute Physiology Score II; SOFA, Sequential Organ Failure Assessment; GCS, Glasgow Coma Scale; CCI, Charlson Comorbidity Index; ICU, Intensive care unit; WBC, White blood cell; RDW, Red blood cell distribution width; eGFR, Estimated glomerular filtration rate; INR, International normalized ratio.

### Association between serum osmolality/BCR with in-hospital mortality of ICH patients

As exhibited in Table 2, age, race, insurance type, marital status, MAP, respiratory rate, urine output-24 h, AKI, SAPSII, SOFA score, GCS score, CCI, ICU type, ventilation, vasopressor, diuretic, mannitol, brain surgery, platelet, RDW, hemoglobin, hematocrit, bicarbonate, and eGFR were potential covariates associated with in-hospital mortality of ICH patients. No significant multicollinearity was observed among these variables, as the generalized variance inflation factors (GVIFs) were all <10 (Supplementary Table 3).

**Table 2:** Univariable Cox regression identifying potential covariates.

Variables	HR (95% CI)	*p*
Age		
<65	Ref	
≥65	1.65 (1.35–2.03)	<0.001
Gender		
Woman	Ref	
Man	0.86 (0.70–1.04)	0.116
Race		
White	Ref	
Black	0.94 (0.66–1.33)	0.714
Other	0.99 (0.72–1.35)	0.932
Unknown	1.53 (1.22–1.93)	<0.001
Insurance		
Medicare	Ref	
Medicaid	0.62 (0.39–0.98)	0.041
Other	0.79 (0.65–0.97)	0.024
Marital status		
Married	Ref	
Single	0.71 (0.53–0.95)	0.023
Divorced	0.75 (0.46–1.24)	0.268
Widowed	1.52 (1.13–2.04)	0.006
Unknown	1.71 (1.34–2.19)	<0.001
MAP	0.99 (0.99–1.00)	0.016
Heart rate	1.00 (1.00–1.01)	0.075
Respiratory rate	1.02 (1.00–1.04)	0.031
Temperature	0.88 (0.77–1.01)	0.070
SpO_2_	1.02 (0.99–1.06)	0.213
PT	1.01 (1.00–1.02)	0.169
Urineoutput-24 h	1.00 (1.00–1.00)	0.004
Cerebrovascular fluid		
No	Ref	
Yes	1.16 (0.92–1.47)	0.220
Hypertension		
No	Ref	
Yes	1.01 (0.81–1.24)	0.950
Neurological deterioration		
No	Ref	
Yes	1.08 (0.87–1.34)	0.475
AKI		
No	Ref	
Yes	1.38 (1.09–1.73)	0.007
Sepsis		
No	Ref	
Yes	1.06 (0.87–1.30)	0.568
SAPSII	1.06 (1.05–1.06)	<0.001
SOFA	1.15 (1.12–1.18)	<0.001
GCS		
<13	Ref	
≥13	0.80 (0.65–0.98)	0.029
CCI	1.04 (1.00–1.09)	0.049
ICU type		
Neuro ICU	Ref	
SICU	1.31 (1.05–1.64)	0.017
Other	1.33 (0.97–1.84)	0.078
Ventilation		
No	Ref	
Yes	2.50 (1.88–3.32)	<0.001
Vasopressor		
No	Ref	
Yes	2.55 (2.06–3.17)	<0.001
Diuretic		
No	Ref	
Yes	1.48 (1.04–2.11)	0.028
Mannitol		
No	Ref	
Yes	2.98 (2.26–3.94)	<0.001
Brain surgery		
No	Ref	
Yes	0.54 (0.32–0.91)	0.020
WBC	1.00 (1.00–1.01)	0.096
Platelet	1.00 (1.00–1.00)	0.002
RDW	1.13 (1.08–1.19)	<0.001
Hemoglobin	0.95 (0.90–0.99)	0.018
Hematocrit	0.98 (0.97–1.00)	0.044
Bicarbonate	0.97 (0.94–0.99)	0.016
eGFR	0.99 (0.98–0.99)	<0.001
INR	1.12 (0.98–1.29)	0.103
Calcium	1.45 (0.90–2.35)	0.129
Chloride	0.99 (0.97–1.01)	0.161

HR, Hazard ratio; CI, Confidence intervals; Ref, Reference; MAP, Mean arterial pressure; SpO_2_, Oxyhemoglobin saturation; PT, Prothrombin time; AKI, Acute kidney injury; SAPSII, Simplified Acute Physiology Score II; SOFA, Sequential Organ Failure Assessment; GCS, Glasgow Coma Scale; CCI, Charlson Comorbidity Index; ICU, Intensive care unit; WBC, White blood cell; RDW, Red blood cell distribution width; eGFR, Estimated glomerular filtration rate; INR, International normalized ratio.

In the univariable Cox regression model, compared to those with serum osmolality of <295 mmol/L, the risk of in-hospital mortality might be increased in those with serum osmolality of ≥295 mmol/L (HR = 1.69, 95%CI: 1.38–2.07). After adjusting for confounding factors, serum osmolality of ≥295 mmol/L was correlated with an increased risk of in-hospital mortality in patients with ICH (HR = 1.43, 95%CI: 1.14–1.78). BCR of >20 might be a risk factor for in-hospital mortality in patients with ICH in the unadjusted model. However, BCR of >20 was not significantly associated with the risk of in-hospital mortality in patients with ICH (Table 3).

**Table 3:** Associations of serum osmolality and BCR with the in-hospital mortality of ICH patients.

Variables	Model 1		Model 2	
	HR (95%CI)	*p*	HR (95%CI)	*p*
Serum osmolality				
<295	Ref		Ref	
≥295	1.69 (1.38–2.07)	<0.001	1.43 (1.14–1.78)	0.002
BCR				
≤20	Ref		Ref	
>20	1.42 (1.16–1.73)	0.001	1.17 (0.95–1.45)	0.142

BCR, Blood uric acid-to-creatinine ratio; Ref, Reference; HR, Hazard ratio, CI, Confidence interval.

Model 1: Univariable Cox regression model.

Model 2: Multivariable Cox regression model adjusted for age, race, insurance, marital status, MAP, respiratory rate, urine output-24 h, AKI, SAPSII, SOFA, GCS, CCI, ICU type, ventilation, vasopressor, diuretic, mannitol, brain surgery, platelet, RDW, hemoglobin, hematocrit, bicarbonate, and eGFR.

### Subgroup analysis of the association of serum osmolality and BCR with the in-hospital mortality of ICH patients

**Figure 2:**
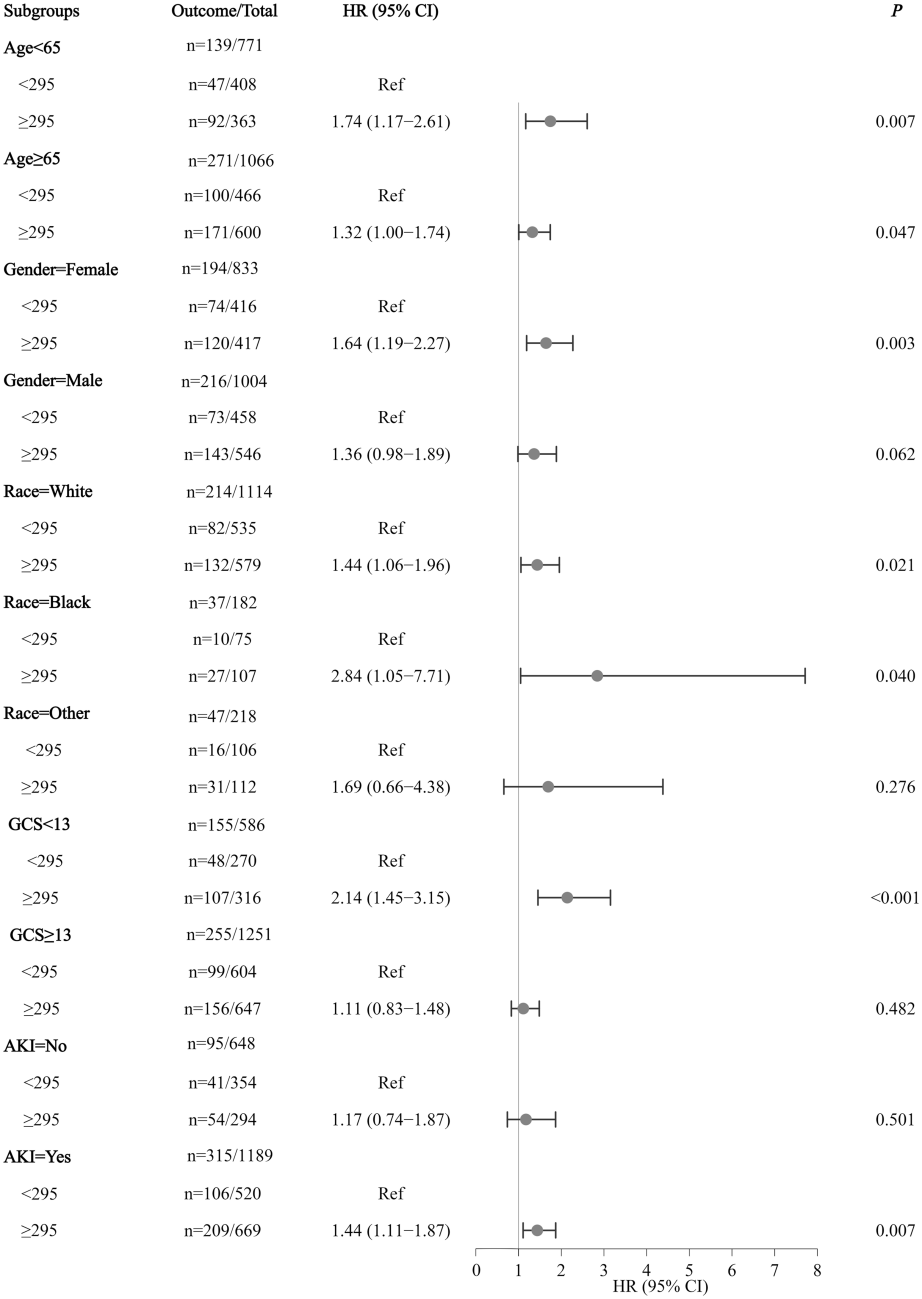
Forest plot of the association between serum osmolality/BCR and in-hospital mortality of ICH patients.

In both participants aged <65 (HR = 1.74, 95%CI: 1.17–2.61) and ≥ 65 (HR = 1.32, 95%CI: 1.00–1.74) years, serum osmolality of ≥295 mmol/L was related to an elevated risk of in-hospital mortality in ICH patients. The increased risk of in-hospital mortality was observed in female ICH patients with serum osmolality of ≥295 mmol/L (HR = 1.64, 95%CI: 1.19–2.27). Serum osmolality of ≥295 mmol/L was correlated with an increased risk of in-hospital mortality of ICH patients who belonged to white (HR = 1.44, 95%CI: 1.06–1.96) or Black (HR = 2.84, 95%CI: 1.05–7.71) race. In ICH patients with a GCS score of ≤13, the elevated risk of in-hospital mortality was found to be associated with serum osmolality of ≥295 mmol/L (HR = 2.14, 95%CI: 1.45–3.15). In ICH patients with AKI complications, we found a heightened risk of in-hospital mortality associated with serum osmolality ≥295 mmol/L (HR = 1.44, 95%CI: 1.11–1.87) (Figure 2).

## Discussion

The current study analyzed the association between serum osmolality and the risk of in-hospital mortality in ICH patients. The results delineated that serum osmolality ≥295 mmol/L was correlated with an increased risk of in-hospital mortality in patients with ICH, while no significant association was observed between BCR >20 and the risk of in-hospital mortality in patients with ICH. The results might offer a reference for the management of ICH patients.

Dehydration upon admission frequently emerges as a significant determinant impacting the anticipated short-term mortality rates in the evaluation of other acute diseases (18). Dehydration upon admission might serve as a significant and independent prognostic indicator for short-term mortality in patients with spontaneous ICH (18). The risk of mortality or dependency at hospital discharge was significantly higher among patients experiencing dehydration compared to those without (26). In a former study based on experience from a tertiary care center, as a dehydration marker, elevation of BCR was reported as a predictor of 30-day mortality among spontaneous ICH patients (27). The BCR is commonly utilized for assessing hydration status; however, it lacks specificity as it may exhibit elevation in patients with gastrointestinal bleeding and other medical conditions (26). In a study conducted by Nag et al., a plasma osmolality of 312 mOsm/kg was observed upon admission, which serves as an additional biomarker for dehydration, exhibiting a significant association with early mortality (within 7 days) and the occurrence of extremely severe strokes, thus serving as an indicator for predicting early mortality (28). High osmolality shortly after admission was found to be significantly associated with mortality in patients with subarachnoid hemorrhage caused by aneurysms (29). These findings potentiate the results in our study, which delineated that serum osmolality of ≥295 mmol/L was correlated with an increased risk of in-hospital mortality in patients with ICH. In a previous study, Gao et al. revealed that dehydration on admission was correlated with reduced in-hospital mortality after ICH (17), which was in contrast with our findings. The possible reasons might be that the study populations were different. Gao et al. explored patients with ICH, while in the present study, ICH patients in the ICU were analyzed. In addition, the dehydration status was evaluated at hospital admission in the study by Gao et al., while in our study, the evaluation of dehydration status was measured at ICU admission. These findings might suggest that the dehydration status of ICH patients might differ in patients with varying degrees of severity.

The mechanisms for the association between dehydration and mortality risk in ICH patients are still unclear. The possible reasons might be that dehydration after stroke was associated with an increased risk of venous thromboembolism and adverse outcomes following hospitalization (30). Dehydration is known to have an impact on reducing cerebral perfusion, decreasing collateral circulation, increasing blood viscosity, and causing hypercoagulability (31). In the future, the investigation and enhanced comprehension of the association between dehydration and in-hospital mortality in patients with ICH necessitate the utilization of biological mechanisms employing animal models as well as prospective cohort studies. This study explored the association of serum osmolality and BCR with the risk of in-hospital death in patients with ICH. Compared to BCR, serum osmolality is considered a more valuable prognostic marker in patients with ICH. BCR serves as a traditional biomarker for assessing dehydration status; however, BCR is not exclusively associated with dehydration and can also show an increase due to other factors. For instance, urea levels may increase in hypercatabolic states such as sepsis, major surgery, or starvation (11). Additionally, a significant increase in BCR can occur with the large “blood protein meal” of an upper gastrointestinal bleed (25) as well as following high-dose glucocorticoid administration (32). Plasma osmolality is the main homeostatic parameter against which humans regulate intracellular hydration.^1^ When individuals have inadequate fluid intake in relation to their fluid losses, there is a decrease in their extracellular fluid volume, while the electrolyte content remains constant. Both osmolality (the concentration of solute particles per kilogram of solvent) and osmolarity (the concentration of solute particles per liter of solution) increase in the absence of excessive electrolyte loss or gain; plasma osmolality can thus generally be used as an indicator for dehydration.

The findings might help screen out more valuable prognostic markers and provide some basis for disease monitoring, treatment decision-making, and prognosis improvement in ICH patients.

Several limitations were found in our study. First, this was a single-center, retrospective study including ICU patients, and there might be some selection bias. Second, only the association between serum osmolality and the risk of in-hospital death in ICH patients was observed, and the causality between serum osmolality and risk of death in ICH patients could not be inferred. Third, variables that might affect the results, such as bleeding location and volume, could not be identified in the database and thus not analyzed in this study. More well-designed studies are required to verify the findings of the current study.

## Conclusion

The association between dehydration status and the risk of in-hospital mortality in ICH patients was assessed in the present study. The findings identified that higher serum osmolality was associated with an increased risk of in-hospital mortality in ICH patients. The findings suggested that serum osmolality holds potential value for the clinical management of ICH patients; therefore, it might be essential to frequently monitor serum osmolality.

## Data availability statement

Publicly available datasets were analyzed in this study. This data can be found here: MIMIC IV database, https://mimic.physionet.org/iv/.

## Ethics statement

Ethical review and approval was not required for the study on human participants in accordance with the local legislation and institutional requirements. Written informed consent from the patients/participants or patients/participants' legal guardian/next of kin was not required to participate in this study in accordance with the national legislation and the institutional requirements.

## Author contributions

ZH: Conceptualization, Data curation, Formal analysis, Investigation, Methodology, Project administration, Supervision, Writing – original draft, Writing – review & editing. QS: Data curation, Formal analysis, Investigation, Methodology, Writing – review & editing.

## Footnotes

^1^ https://nap.nationalacademies.org/read/10925/chapter/1
