# Supplementary material for: Association between serum osmolality and risk of in-hospital mortality in patients with intracerebral hemorrhage
Source: Front Neurol. 2024 Aug 2;15:1410569. doi: 10.3389/fneur.2024.1410569 (PMC11327125; doi:10.3389/fneur.2024.1410569)
Supplement: Supplementary file 1 [file Table_1.DOCX]

Supplementary Table 1 The status and management of the missing vales

| Variables | n | % | Management |
| --- | --- | --- | --- |
| Heart rate | 1 | 0.05% | Multiple imputation |
| SpO_2_ | 1 | 0.05% | Multiple imputation |
| Temperature | 5 | 0.27% | Multiple imputation |
| WBC | 10 | 0.54% | Multiple imputation |
| Hematocrit | 10 | 0.54% | Multiple imputation |
| Platelet | 11 | 0.6% | Multiple imputation |
| RDW | 11 | 0.6% | Multiple imputation |
| Hemoglobin | 11 | 0.6% | Multiple imputation |
| Respiratory rate | 78 | 4.25% | Multiple imputation |
| Calcium | 85 | 4.63% | Multiple imputation |
| PT | 206 | 11.21% | Multiple imputation |
| INR | 206 | 11.21% | Multiple imputation |

SpO_2_: Oxyhemoglobin saturation; WBC: White blood cell; RDW: Red blood cell distribution width; PT: Prothrombin time; INR: International normalized ratio.

Supplementary Table 2 Sensitivity analysis comparing the data before and after imputation

| Variables | Before imputation (n=1837) | After imputation (n=1837) | Statistics | *P* |
| --- | --- | --- | --- | --- |
| Heart rate, bpm, Mean (±SD) | 82.46 (±17.63) | 82.47 (±17.64) | t = -0.022 | 0.983 |
| SpO_2_, %, M (Q₁, Q₃) | 98 (96-100) | 98 (96-100) | W = 1686558 | 0.995 |
| Temperature, ℃, Mean (±SD) | 36.84 (±0.67) | 36.84 (±0.67) | t = -0.012 | 0.990 |
| WBC, K/uL, M (Q₁, Q₃) | 10.2 (7.9-13.1) | 10.2 (7.9-13.1) | W = 1675985 | 0.947 |
| Hematocrit, %, Mean (±SD) | 36.30 (±5.83) | 36.31 (±5.82) | t = -0.009 | 0.993 |
| Platelet, K/uL, Mean (±SD) | 213.13 (±85.35) | 213.13 (±85.12) | t = 0.000 | 1.000 |
| RDW, %, Mean (±SD) | 14.21 (±1.67) | 14.21 (±1.67) | t = -0.003 | 0.997 |
| Hemoglobin, g/dL, Mean (±SD) | 12.08 (±2.04) | 12.08 (±2.04) | t = 0.015 | 0.988 |
| Respiratory rate, insp/min, Mean (±SD) | 18.51 (±5.28) | 18.51 (±5.21) | t = 0.019 | 0.985 |
| Calcium, mg/dL, Mean (±SD) | 0.54 (±0.17) | 0.53 (±0.17) | t = 0.055 | 0.956 |
| PT, sec, M (Q₁, Q₃) | 12.6 (11.6-14.1) | 12.68 (11.7-14) | W = 1471325 | 0.363 |
| INR, M (Q₁, Q₃) | 1.1 (1.1-1.3) | 1.12 (1.1-1.3) | W = 1475768.5 | 0.439 |

SD: Standard Deviation; M: Median; Q₁: 1st Quartile; Q₃: 3st Quartile; t: Student's t test; t': Satterthwaite t test; W: Wilcoxon rank sum test; χ²: Chi-square test; -: Fisher's exact test; SpO_2_: Oxyhemoglobin saturation; WBC: White blood cell; RDW: Red blood cell distribution width; PT: Prothrombin time; INR: International normalized ratio.

Supplementary Table 3 The evaluation of potential collinearity of variables

| Variables | GVIF |
| --- | --- |
| Age | 1.5529 |
| Race | 2.4108 |
| Insurance | 1.4075 |
| Marital status | 2.4063 |
| MAP | 1.2154 |
| Respiratory rate | 1.0995 |
| 24 h urine output | 1.4474 |
| AKI | 1.1616 |
| SAPSII | 2.3729 |
| SOFA | 3.1781 |
| GCS | 1.4235 |
| CCI | 1.3241 |
| ICU type | 1.7848 |
| Ventilation | 1.1491 |
| Vasopressor | 1.7537 |
| Diuretic | 1.2687 |
| Mannitol | 1.3308 |
| Brain surgery | 1.0767 |
| Platelet | 1.2378 |
| RDW | 1.4665 |
| Hemoglobin | 2.2622 |
| Hematocrit | 2.5778 |
| Bicarbonate | 1.2750 |
| eGFR | 1.6653 |

GVIF: generalized variance inflation factor; MAP: Mean arterial pressure; KI: Acute kidney injury; SAPSII: Simplified acute physiology score II; SOFA: Sequential organ failure assessment; GCS: Glasgow coma scale; CCI: Charlson comorbidity index; ICU: Intensive care unit; RDW: Red blood cell distribution width; eGFR: estimated glomerular filtration rate
